# Circulatory Disturbances in Traumatic Lesions of the Brain

**Published:** 1876-06

**Authors:** 


The Circulatory Disturbances Visible to the Ophthalmoscope in Traumatic Lesions of the Brain.—M. Panas concluded a memoir on this subject, read before the Academie de Medecin, with the following resume of facts, that were established by his observations:

1. Papillary stasis often occurs as a result of the different traumatic lesions of the encephalon (concussion, contusion, wounds, fractures of the cranium, etc.)

2. This stasis in question is not always accompanied by visual disturbances; consequently the ophthalmoscope should be employed in all cases of injury to the head, whether a diminution in the acuity of vision be complained of or not.

3. According to the autopsies made, and according to Schwalbe, this stasis seems to depend on an infiltration of blood or of serum into the sheath of the optic nerve, and is not directly due to the cerebral lesion.

4. The papillary stasis cannot be said to belong to one variety of cerebral lesion rather than to another, nor does it afford us any assistance in judging of the gravity of the lesion.

5. All that can be affirmed in the present state of our knowledge, is that the papillary stasis indicates the presence of an effusion into the meninges.—*La France Medicale.*

